# A Three-Dimensional Cell Culture Model To Study Enterovirus Infection of Polarized Intestinal Epithelial Cells

**DOI:** 10.1128/mSphere.00030-15

**Published:** 2015-11-18

**Authors:** Coyne G. Drummond, Cheryl A. Nickerson, Carolyn B. Coyne

**Affiliations:** a Department of Microbiology and Molecular Genetics, University of Pittsburgh, Pittsburgh, Pennsylvania, USA; b School of Life Sciences, Arizona State University, Tempe, Arizona, USA; c The Biodesign Institute, Center for Infectious Diseases and Vaccinology, Arizona State University, Tempe, Arizona, USA; Boston University School of Medicine

**Keywords:** intestinal epithelial cell, enterovirus, coxsackievirus B, 3-D cell culture, RWV bioreactor

## Abstract

Despite serving as the primary entry portal for coxsackievirus B (CVB), little is known about CVB infection of the intestinal epithelium, owing at least in part to the lack of suitable *in vivo* models and the inability of cultured cells to recapitulate the complexity and structure associated with the gastrointestinal (GI) tract. Here, we report on the development of a three-dimensional (3-D) organotypic cell culture model of Caco-2 cells to model CVB infection of the gastrointestinal epithelium. We show that Caco-2 cells grown in 3-D using the rotating wall vessel (RWV) bioreactor recapitulate many of the properties of the intestinal epithelium, including the formation of well-developed tight junctions, apical-basolateral polarity, brush borders, and multicellular complexity. In addition, transcriptome analyses using transcriptome sequencing (RNA-Seq) revealed the induction of a number of genes associated with intestinal epithelial differentiation and/or intestinal processes *in vivo* when Caco-2 cells were cultured in 3-D. Applying this model to CVB infection, we found that although the levels of intracellular virus production were similar in two-dimensional (2-D) and 3-D Caco-2 cell cultures, the release of infectious CVB was enhanced in 3-D cultures at early stages of infection. Unlike CVB, the replication of poliovirus (PV) was significantly reduced in 3-D Caco-2 cell cultures. Collectively, our studies show that Caco-2 cells grown in 3-D using the RWV bioreactor provide a cell culture model that structurally and transcriptionally represents key aspects of cells in the human GI tract and can thus be used to expand our understanding of enterovirus-host interactions in intestinal epithelial cells.

**IMPORTANCE** Coxsackievirus B (CVB), a member of the enterovirus family of RNA viruses, is associated with meningitis, pericarditis, diabetes, dilated cardiomyopathy, and myocarditis, among other pathologies. CVB is transmitted via the fecal-oral route and encounters the epithelium lining the gastrointestinal tract early in infection. The lack of suitable *in vivo* and *in vitro* models to study CVB infection of the gastrointestinal epithelium has limited our understanding of the events that surround infection of these specialized cells. Here, we report on the development of a three-dimensional (3-D) organotypic cell culture model of human intestinal epithelial cells that better models the gastrointestinal epithelium *in vivo*. By applying this 3-D model, which recapitulates many aspects of the gastrointestinal epithelium *in vivo*, to the study of CVB infection, our work provides a new cell system to model the mechanisms by which CVB infects the intestinal epithelium, which may have a profound impact on CVB pathogenesis.

**Podcast**: A podcast concerning this article is available.

## Abstract (precis)

Coxsackievirus B (CVB), a member of the enterovirus family of RNA viruses, is associated with meningitis, pericarditis, diabetes, dilated cardiomyopathy, and myocarditis, among other pathologies. CVB is transmitted via the fecal-oral route and encounters the epithelium lining the gastrointestinal tract early in infection. The lack of suitable *in vivo* and *in vitro* models to study CVB infection of the gastrointestinal epithelium has limited our understanding of the events that surround infection of these specialized cells. Here, we report on the development of a three-dimensional (3-D) organotypic cell culture model of human intestinal epithelial cells that better models the gastrointestinal epithelium *in vivo*. By applying this 3-D model, which recapitulates many aspects of the gastrointestinal epithelium *in vivo*, to the study of CVB infection, our work provides a new cell system to model the mechanisms by which CVB infects the intestinal epithelium, which may have a profound impact on CVB pathogenesis.

## INTRODUCTION

Enteroviruses, small, positive-strand, single-stranded RNA (ssRNA) viruses of the *Picornaviridae* family, are primarily transmitted by the fecal-oral route and encounter the epithelium lining the gastrointestinal (GI) tract early in infection. Intestinal epithelial cells (IECs) are formidable barriers to pathogen entry, owing in part to the highly differentiated and complex nature of their apical surfaces, which are composed of rigid densely packed microvilli coated with a mucin-enriched glycocalyx, and the presence of junctional complexes between cells that restrict pathogen access to the interstitial space. In addition to the barrier presented by enterocytes themselves, the multicellular nature of the GI epithelium, which is composed of goblet cells, Paneth cells, and Microfold (M) cells, the latter of which are found in Peyer’s patches, also serve to restrict pathogen entry. Little is known regarding the events that surround enterovirus infection of the GI tract owing at least in part to the lack of suitable *in vivo* models for the enteric entry route of these viruses and to the inability of standard cultured cells to recapitulate the complexity and structure associated with the gastrointestinal epithelium.

The lack of enterovirus infection following oral administration in mice has been attributed to the inability of many of these viruses to bind to the murine homologs of their entry receptors and/or attachment factors (1–3). However, poliovirus (PV) replicates inefficiently in mice expressing the human poliovirus receptor (PVR) and exhibits higher levels of replication when the type I interferon (IFN) system is ablated by deletion of the alpha/beta interferon (IFN-α/β) receptor (4). Similarly, expression of human decay-accelerating factor (DAF) (also known as CD55), which serves as an attachment factor for coxsackievirus B3 (CVB) (2, 5) and is required for apical infection of cultured enterocytes (6), is also not sufficient to mediate high levels of viral replication when the virus is delivered by the enteral route, which occurs only upon IFN-α/β receptor deletion (7). In addition, although murine models have been developed for both CVB-induced pancreatitis (8, 9) and cardiomyopathy (10, 11), these models require intraperitoneal infection, thus bypassing IECs as an infection barrier.

Based upon cell culture models, there are several key differences between the mechanisms by which CVB infects polarized IECs and nonpolarized cells, such as HeLa cells. The polarized nature of IECs poses an inherent complexity for CVB entry. CVB utilizes DAF as an apical attachment factor and requires delivery of apically bound viral particles to the tight junction (TJ) complex to interact with its entry receptor, the coxsackievirus and adenovirus receptor (CAR) (12, 13). In polarized IECs, CVB accomplishes this through hijacking the cytoskeleton and inducing intracellular tyrosine family kinase signaling, which results in virus delivery to the TJ and eventual access to the cytoplasm by caveola- and macropinocytosis-associated pathways (13, 14). In nonpolarized cells, CAR is readily accessible to viral particles and does not require DAF for attachment or entry (6). Accordingly, the mechanism of entry differs dramatically from that for IECs (15). Postentry, CVB replication is also facilitated by IEC-specific factors (16), and CVB egress from IECs is mediated by a different cell death pathway from that observed in nonpolarized cells (17). Collectively, these previous studies have pointed to important differences in the life cycle of CVB between polarized IECs and nonpolarized cells and suggest that these differences play important roles in viral pathogenesis.

Although the use of cultured intestinal cells has provided the foundation for much of what we know about CVB infection of polarized IECs, an inherent limitation of these cell systems is their inability to recapitulate the architecture and multicellular complexity associated with the human GI tract. Culturing many enteric cell lines in three dimensions (3-D) has provided an excellent model system to mimic the morphological and/or functional features of these cells *in vivo* and to better model their susceptibility to microorganisms (reviewed in reference  18). The rotating wall vessel (RWV) bioreactor, which was initially developed by NASA to recapitulate aspects of the quiescent microgravity environment, has emerged as an advantageous method to culture cells in 3-D, as it recapitulates physiologically relevant, low levels of shear and turbulence (18–20). Enteric cell lines cultured in this system exhibit many characteristics normally associated with fully differentiated functional IECs *in vivo*, including distinct apical and basolateral polarity, increased expression and better organization of TJs, enhanced expression of brush border proteins, and highly localized expression of mucins, and they also exhibit multicellular complexity (including the presence of M cells or M-like cells [M/M-like cells], goblet cells, Paneth cells, and enterocytes), which does not occur using standard two-dimensional (2-D) culture systems (18, 21–26). Enterocytes cultured in this system also display important differences from 2-D cultured cells with respect to their susceptibility to bacterial attachment and invasion. For example, *Salmonella enterica* serovar Typhimurium exhibits reductions in its ability to adhere to IECs grown in 3-D (27) and exhibits reduced invasion in IECs cultured in 3-D (22, 27). In addition to intestinal models, the structural complexity of other cell types grown in 3-D has resulted in the development of infection models for a diverse array of pathogens and tissue types (reviewed in reference 18), including hepatitis C virus in hepatocytes (28), *Pseudomonas aeruginosa*, and *Francisella tularensis* in the alveolar epithelium (29), and HIV in lymphoid tissue (30).

Given the lack of suitable *in vivo* models of CVB enteric infection, we utilized the RWV bioreactor to develop a 3-D culture system of human IECs to better model their infection by CVB. Caco-2 cells were chosen as the cell type to use in this model, given that they have previously served as a cell culture model for CVB infection of IECs *in vitro* (13, 14, 16, 17) and have been used previously in the RWV bioreactor (31, 32). We found that Caco-2 cells cultured in 3-D using the RWV bioreactor displayed morphological and transcriptional changes more similar to the GI epithelium *in vivo*. Strikingly, we found by transcriptome sequencing (RNA-Seq) analyses that Caco-2 cells cultured in 3-D robustly express transmembrane mucins that form the enterocyte apical glycocalyx and specific markers of goblet and enterocyte cell differentiation, whereas these transcripts are not expressed or are expressed at low levels in 2-D cultures. In addition, we show that Caco-2 cells grown in 3-D are susceptible to CVB infection, but they produce lower levels of viral RNA (vRNA) and newly synthesized viral protein than cells cultured in 2-D. However, despite the lower levels of vRNA and viral protein, we found that intracellular titers of CVB were similar between 2-D and 3-D cultures. Interestingly, we also found that CVB was released into the medium of infected Caco-2 cells cultured in 3-D more efficiently at earlier time points than what was observed in 2-D cultured cells, suggesting that viral release may occur with greater efficiency in this model. Given the significant morphological changes and changes in expression induced in Caco-2 cells grown in 3-D, and their susceptibility to CVB infection, this system can be used to better model the interaction of CVB, and possibly other viruses, with polarized IECs.

## RESULTS

### Establishment of 3-D cultures of Caco-2 cells using the RWV bioreactor.

**FIG 1:**
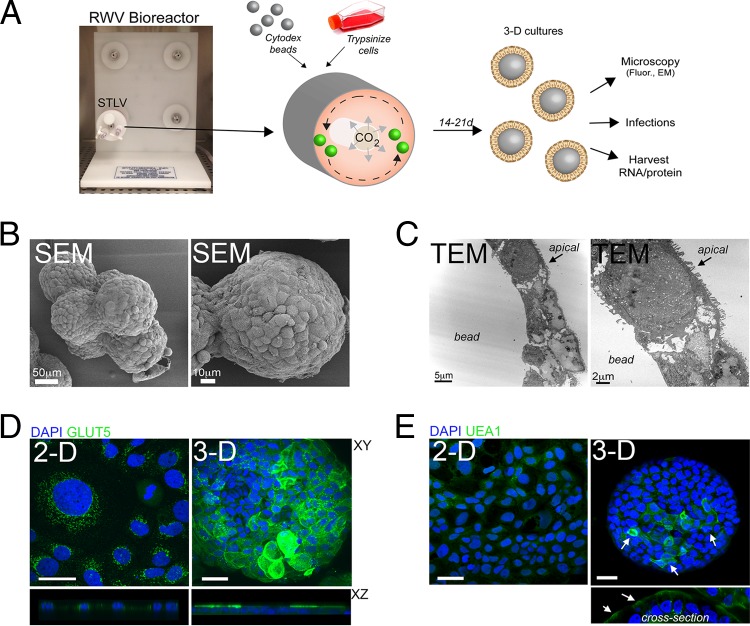
(A) Schematic for the culturing of cells in the RWV bioreactor. The slow-turning lateral vessel (STLV) is shown. Green spheres in the schematic represent cell-coated Cytodex beads. The cells were cultured for 14 to 21 days (14-21d). (B) Scanning electron micrograph (SEM) of Caco-2 cells cultured in the RWV bioreactor for 21 days. (C) Transmission electron microscopy (TEM) of Caco-2 cells cultured in the RWV bioreactor for 21 days. The black arrows denote the apical surface. (D) Confocal microscopy for GLUT5 (green) in 2-D or 3-D cultures of Caco-2 cells. The *x*-*y* image (top) and the *x*-*z* cross section (bottom) are shown. DAPI-stained nuclei are shown in blue. (E) Confocal microscopy for UEA1 (green) in 2-D or 3-D Caco-2 cell cultures. The white arrows denote specific sites of fluorescence in 3-D beads. A cross section of 3-D culture is shown at the bottom. DAPI-stained nuclei are shown in blue. Bars, 10 µm (D and E).

The RWV bioreactor consists of slow-turning lateral vessels (STLVs), which are completely filled with cell culture medium and contain cells attached to porous, extracellular matrix (ECM)-coated beads (or other scaffolds) (19) (schematic in Fig. 1A). STLVs are kept in constant rotation by a powered apparatus, allowing for cells and beads to remain in perpetual suspension. We established this system for Caco-2 cells using collagen-coated porous dextran beads (Cytodex-3) and cultured cells for a period of 21 days prior to their removal from the STLVs and subsequent processing for downstream applications (schematic in Fig. 1A). We found that Caco-2 cells fully coated Cytodex beads during the culture period and formed complete, uniform single layers of cells and organoids composed of cell-bead aggregates as assessed by both scanning electron microscopy (SEM) and transmission electron microscopy (TEM) (Fig. 1B and C).

Several GI-derived cell lines, including HT-29 and INT-407 cells, develop multicellular complexity (including the presence of M/M-like cells, goblet cells, Paneth cells, and enterocytes) when cultured in 3-D (18, 21–26). To assess the differentiation of RWV-cultured Caco-2 cells, we performed fluorescence confocal microscopy for markers of IEC subtypes. We used an antibody directed against GLUT5, a fructose transporter of the SLC2 family which localizes to the lumen of human enterocytes (33) and fluorescein thiocyanate (FITC)-conjugated lectin, *Ulex europaeus* agglutinin I (UEA1), which binds l-fructose present in intestinal mucin and is associated with M/M-like (34) and goblet cells (35). We found a pronounced enhancement of GLUT5 immunofluorescence in 3-D versus 2-D cultured Caco-2 cells, which correlated with the pronounced redistribution of GLUT5 from intracellular punctae in 2-D cultures to the apical surface in 3-D cultured cells (Fig. 1D). Similarly, we found that UEA1 was more abundantly expressed on 3-D cultured Caco-2 cells and exhibited an apical localization, consistent with its *in vivo* localization (36) (Fig. 1E).

### 3-D Caco-2 cell cultures develop cell-cell junctions and brush borders.

**FIG 2:**
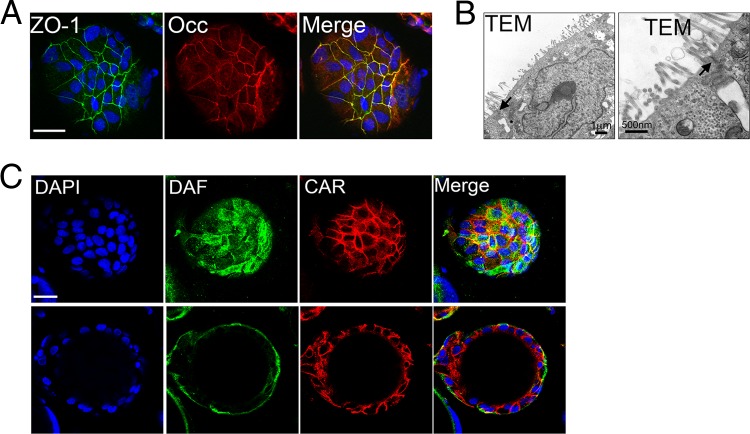
(A) Confocal microscopy for ZO-1 (green) and occludin (red) in 3-D Caco-2 cells cultured for 21 days. DAPI-stained nuclei are shown in blue. (B) Transmission electron micrographs of 3-D cultures of Caco-2 cells. The black arrows denote the junctional complex between cells. (Right) Zoomed-in image of the image shown to the left. (C) Confocal microscopy for DAF (green) and CAR (red) in 3-D Caco-2 cells cultured for 21 days. DAPI-stained nuclei are shown in blue. View of the surface of a bead (top) and cross-sectional view of the same bead (bottom) are shown. Bars, 10 µm (A and C).

The polarization of IECs protects the interstitial tissue of the lamina propria from foreign substances and pathogens in the intestinal lumen. The integrity of the epithelium as a barrier to microbial infection depends on properly formed cell-to-cell junctions, which include the apical-most TJ complex. We found that 3-D cultures of Caco-2 cells developed well-formed TJs, as assessed by the localization of the TJ-associated proteins ZO-1 and occludin to cell-cell borders (Fig. 2A). The presence of cellular junctions was confirmed by TEM, which revealed the presence of adjoining membranes between neighboring cells in 3-D cultured cells at the apical-most domain of the paracellular space (Fig. 2B).

The TJs of IECs present an initial barrier to CVB entry, as CAR, the viral receptor required for CVB uncoating, is localized within these junctions and is inaccessible to the virus from the apical surface. CVB can access CAR and internalize only after cytoskeletal rearrangements that follow viral binding to the apical viral attachment factor DAF (11). Therefore, for an IEC model of CVB infection to accurately portray the mechanism of CVB entry, proper localization of CAR and DAF are required. We confirmed the asymmetric distribution of the CVB attachment factor DAF to the apical surface and CAR to the junctional complex of 3-D Caco-2 cell cultures (Fig. 2C), which also occurs in 2-D cultures (13).

**FIG 3:**
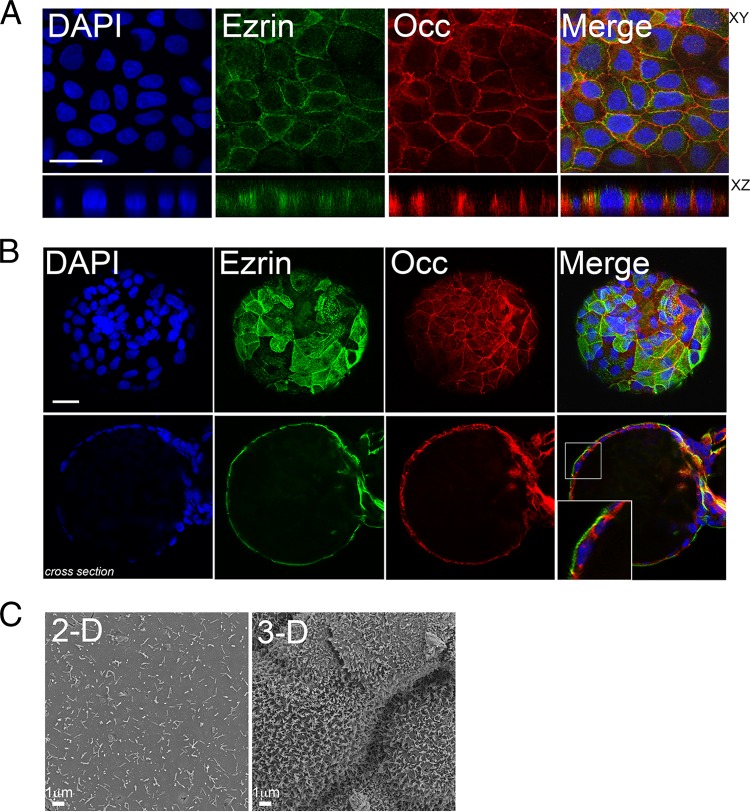
(A) Confocal microscopy for ezrin (green) and occludin (red) in 2-D cultures of Caco-2 cells. DAPI-stained nuclei are shown in blue. The *x*-*y* image (top) and the *x*-*z* image (bottom) are shown. (B) Confocal microscopy for ezrin (green) and occludin (red) in 3-D Caco-2 cells. DAPI-stained nuclei are shown in blue. View of the surface of a bead (top) and cross-sectional view of the same bead (bottom) are shown. (C) Scanning electron micrographs of 2-D or 3-D cultures of Caco-2 cells. Bars, 10 µm (A and B).

The differentiation of IECs to form well-developed brush borders constitutes a major barrier to pathogen infection from the apical surface. To explore the differences in cell differentiation in Caco-2 cells grown in 2-D versus 3-D, we assessed the development of brush borders by immunofluorescence microscopy for ezrin, a member of the ERM family (ezrin, radixin, and moesin) that localizes to microvilli (37), and by SEM. We observed a pronounced difference in ezrin localization between Caco-2 cells grown in 2-D versus 3-D, whereas ezrin was primarily localized to cell junctions in cells grown in 2-D (Fig. 3A), it localized heavily and almost exclusively to the apical surfaces of Caco-2 cells cultured in 3-D (Fig. 3B). These results were corroborated by SEM, which revealed major differences in the development of brush borders between cells grown in 2-D versus 3-D, with 3-D cultures exhibiting the typical thin, “finger-like” projections of microvilli at their apical surfaces (Fig. 3C).

### Transcriptional profiling of 2-D versus 3-D cultures of Caco-2 cells by RNA-Seq.

**FIG 4:**
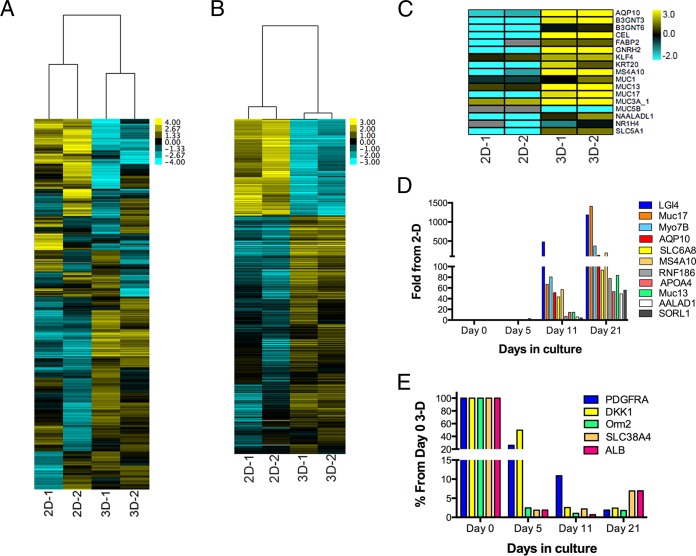
(A) Hierarchical clustering heat map of genes expressed in 2-D or 3-D cultures of Caco-2 cells as determined by RNA-Seq. (B) Hierarchical clustering heat map of genes differentially expressed (*P* < 0.001) in 2-D or 3-D cultures of Caco-2 cells as determined by RNA-Seq, followed by DeSeq2 analysis. (C) Heat map of select markers of intestinal differentiation and/or intestine-specific processes in 2-D or 3-D cultures of Caco-2 cells. The intensity of the color in panels A to C indicates the level of gene expression (yellow for upregulation and blue for downregulation), and gray indicates that no RNA-seq reads were detected for that transcript in that sample. RNA-Seq was performed on two independent 2-D cultures (2D-1 and 2D-2) and two independent 3-D STLVs (3D-1 and 3D-2). (D and E) RT-qPCR analysis of genes upregulated (D) or downregulated (E) in 3-D cultures of Caco-2 cells. In panel D, data are shown as the fold change in the expression of the indicated genes relative to the level of expression in 2-D controls at the indicated days postculturing in 3-D. In panel E, data are shown as the percent change in the expression of the indicated genes relative to the levels at day 0 of the 3-D culture period.

To extend the morphological differences between 2-D and 3-D cultures of Caco-2 cells described above to the transcriptome, we performed RNA-Seq analyses to determine global transcriptional changes that occur as a result of culturing Caco-2 cells in 3-D. We observed significant changes in gene expression when Caco-2 cells were cultured in 3-D cultures compared to 2-D control cultures (Fig. 4A). To identify genes whose expression was significantly different upon culturing cells in 3-D, we performed differential expression analysis using DeSeq2 (38). We identified 1,596 genes (*P* < 0.001) that were differentially expressed in 2-D and 3-D cultures of Caco-2 cells (Fig. 4B; see Data Set S1 in the supplemental material). Interestingly, many of the most upregulated genes in 3-D cultures are associated with intestinal differentiation and/or play specific roles in intestinal processes (Fig. 4C). These genes included gonadotropin-releasing hormone 2 (GnRH2), which was the most differentially upregulated gene in 3-D cultures and is expressed primarily in the small intestine (39), the transmembrane mucins MUC1, MUC13, and MUC17, which are abundantly expressed in the intestine *in vivo* (36, 40) and form the enterocyte apical glycocalyx, and the duodenum and jejunum-associated aquaporin AQP10 (41). In addition, *N*-acetyllactosaminide β-1,3-*N*-acetylglucosaminyltransferase 3 and 6 (B3GNT3 and B3GNT6), which are involved in glycan regulation, the goblet cell-specific differentiation factor KLF4 (42), and cytokeratin 20 (KRT20), a specific marker of intestinal differentiation (43), were all significantly upregulated in 3-D Caco-2 cultures (Fig. 4C). Significantly downregulated genes included the platelet-derived growth factor family member PDGFRA (platelet-derived growth factor receptor alpha), the protease-activated transporter SLC10A4, and Dickkopf Wnt signaling pathway inhibitor 1 (DKK1).

10.1128/mSphere.00030-15.1 Data Set S1  Spreadsheet of DeSeq2 analysis of RNA-Seq studies of 2-D and 3-D cultures of Caco-2 cells. The gene symbols, log_2_ fold changes (green), *P* values (red), and unique gene reads for two 2-D samples (gray) and two 3-D (purple) samples are shown. Download Data Set S1, XLSX file, 0.2 MB. Copyright © 2015 Drummond et al. 2015 Drummond et al. This content is distributed under the terms of the Creative Commons Attribution 4.0 International license.

To confirm the results of our RNA-Seq studies and to determine the kinetics by which these genes were differentially regulated over the culture period of cells grown in 3-D, we performed reverse transcription-quantitative PCR (RT-qPCR) on a panel of the most upregulated and downregulated genes, using RNA extracted from an independent Caco-2 STLV culture at days 5, 11, and 21 after seeding, as well as from Caco-2 cells prior to STLV seeding (day 0). Analyzing the expression of 11 upregulated genes over the course of 3-D culturing, not only did we confirm our RNA-Seq results, we found that in all cases, the induction of these genes occurred between days 11 and 21 of the culture period (Fig. 4E). We found by profiling the expression of five representative downregulated genes that while some genes, such as Orm2, SLC38A4, and ALB, were downregulated early (between days 0 and 5) following the initiation of 3-D culturing, others (PDGFRA and DKK1) became downregulated at later stages (after day 5) of 3-D culturing. These results highlight the profound transcriptome differences between cells cultured in 2-D versus 3-D and suggest that the alterations in gene expression occur at various stages of the culture period.

### Coxsackievirus B infection in 2-D versus 3-D cell cultures of Caco-2 cells.

**FIG 5:**
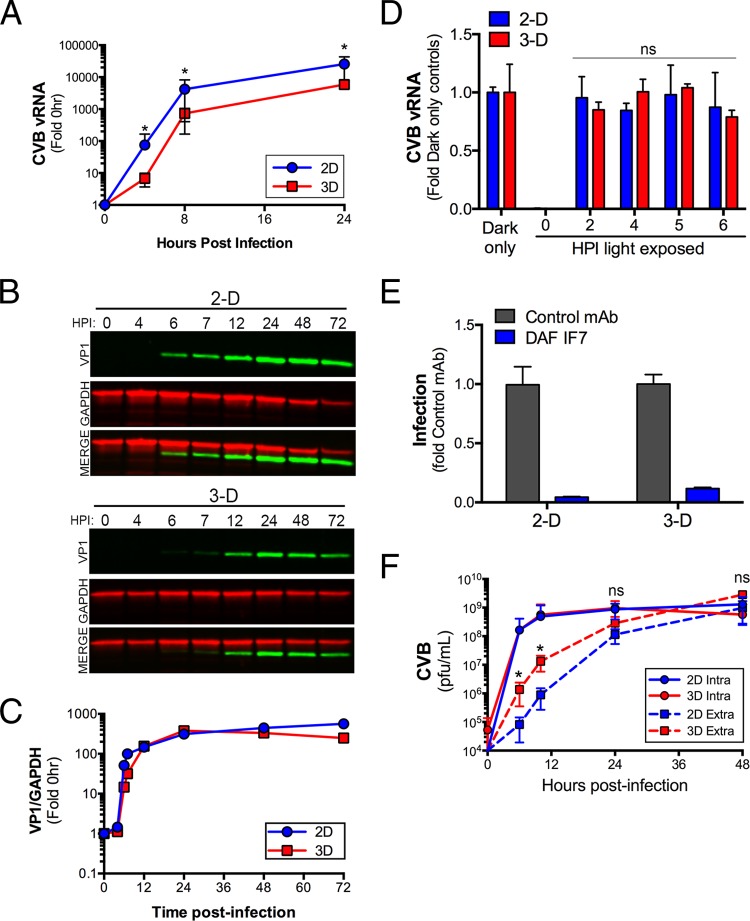
(A) RT-qPCR analyses of vRNA levels of 2-D and 3-D cultures of Caco-2 cells infected with CVB (10 PFU/cell) at the indicated hour postinfection (p.i.). Data are shown as fold change from 0 h p.i. (B) LICOR immunoblots for VP1 (green) and GAPDH (red) from 2-D and 3-D cultures of Caco-2 cells infected with CVB (10 PFU/cell) at the indicated hour postinfection (HPI). Representative data from a single (of three total) STLVs are shown. (C) Densitometry from immunoblots shown in panel B. Data are shown as the levels of VP1 normalized to the level of GAPDH at the indicated times p.i. (D) RT-qPCR analyses of CVB vRNA from 2-D or 3-D cultures of Caco-2 cells infected with light-sensitive neutral red (NR)-containing CVB (1 PFU/cell) and exposed to light at the indicated hours postinfection (HPI). In parallel, cultures were infected with NR-CVB in the dark. Data are shown as fold change from dark-only control infections. (E) RT-qPCR analyses of CVB vRNA from 2-D or 3-D cultures of infected Caco-2 cells or cells pretreated with a control monoclonal antibody (mAb) or anti-DAF IF7 blocking monoclonal antibody. Data are shown as fold change (mean plus standard deviation [error bar]) from the level with the control MAb. (F) CVB titers (in PFU per milliliter) of virus collected from the medium (extracellular [Extra]) (dashed lines) of 2-D or 3-D cultures of Caco-2 cells infected with CVB for the indicated hours postinfection. In addition, CVB titers from cells (intracellular [Intra]) (solid lines) from CVB-infected 2-D or 3-D cultures are shown. Data in panel A and panels D to F are shown as means plus standard deviations and are averaged from three (A and F) or two (D and E) independent STLVs. Values that are significantly different (*P* < 0.05) by a Student’s *t* test are indicated by an asterisk. Values that are not significantly different (ns) as determined by a Student’s *t* test are also indicated.

Given that we observed significant differences in the morphology and transcriptional profiles of cells grown in 2-D versus 3-D, we next assessed whether Caco-2 cells grown in 3-D would exhibit any differences in their susceptibility to CVB infection. To do this, we assessed the levels of CVB vRNA, protein, and infectious virus production over a period of 24 to 72 h postinfection (p.i.). We found that Caco-2 cells grown in 2-D produced significantly more CVB vRNA than did cells grown in 3-D at all time points tested (between 4 and 24 h p.i.) (Fig. 5A). In addition, we found that there was a slight delay in the appearance of newly synthesized viral protein, as assessed by immunoblotting for the CVB capsid protein VP1, in Caco-2 cells grown in 3-D, and less overall VP1 produced at very late stages of infection (72 h p.i.) (Fig. 5B and C).

To determine whether the initial lag in CVB replication observed in 3-D cell cultures was due to a delay in viral internalization, we performed a neutral red (NR) infection assay. By propagating CVB in the presence of the RNA binding dye NR, the virus becomes sensitive to light, which is reversed upon viral uncoating and diffusion of NR away from the vRNA (44). We observed equivalent levels of light sensitivity of NR-CVB between 2-D and 3-D cultures at 0 h p.i., which was lost in both culture conditions by 2 h p.i., indicating that uncoating had occurred in both 2-D and 3-D cultures by 2 h p.i. (Fig. 5D). This is consistent with previous work demonstrating that CVB undergoes uncoating between 90 and 120 min p.i. in Caco-2 cells in 2-D (13). In addition, similar to previous work in 2-D Caco-2 models (6), we found that DAF was required for CVB infection of Caco-2 cells in 3-D given that infection was inhibited in both 2-D and 3-D cultures by a monoclonal anti-DAF antibody that blocks CVB binding (Fig. 5E). Importantly, the levels of CAR and DAF were near equivalent in 2-D and 3-D cultures as assessed by RNA-Seq; therefore, receptor expression does not impact infection levels (see Fig. S1 in the supplemental material).

10.1128/mSphere.00030-15.2 Figure S1  (A) Unique gene reads and reads per kilobase of transcript per million mapped reads (RPKM) values from 2-D and 3-D cultures of Caco-2 cells for CAR (CXADR), DAF (CD55), and PVR. (B) Heat map of log_2_-transformed RPKM values from 2-D and 3-D Caco-2 cell cultures. The intensity of the color indicates the level of gene expression (yellow for high expression and blue for low expression), and gray indicates that no RNA-Seq reads were detected for that transcript in that sample. Download Figure S1, TIF file, 0.1 MB. Copyright © 2015 Drummond et al. 2015 Drummond et al. This content is distributed under the terms of the Creative Commons Attribution 4.0 International license.

We next profiled the levels of CVB replication in 2-D and 3-D Caco-2 cell cultures by measuring intracellular and extracellular infectious virus titers between 0 and 48 h p.i. We found that, whereas intracellular titers of CVB were near equivalent between 2-D and 3-D Caco-2 cell cultures at all time points tested, there was a substantial enhancement in the release of infectious CVB from cells cultured in 3-D at early time points (6 to 12 h p.i.) (Fig. 5F). Taken together, these data show that CVB enters and infects Caco-2 cells grown in 3-D and can be released from cells cultured in 3-D with greater efficiency at early time points of infection compared to cells cultured in 2-D.

### 2-D and 3-D cultures of Caco-2 cells exhibit similar levels of cell death in response to CVB infection.

**FIG 6:**
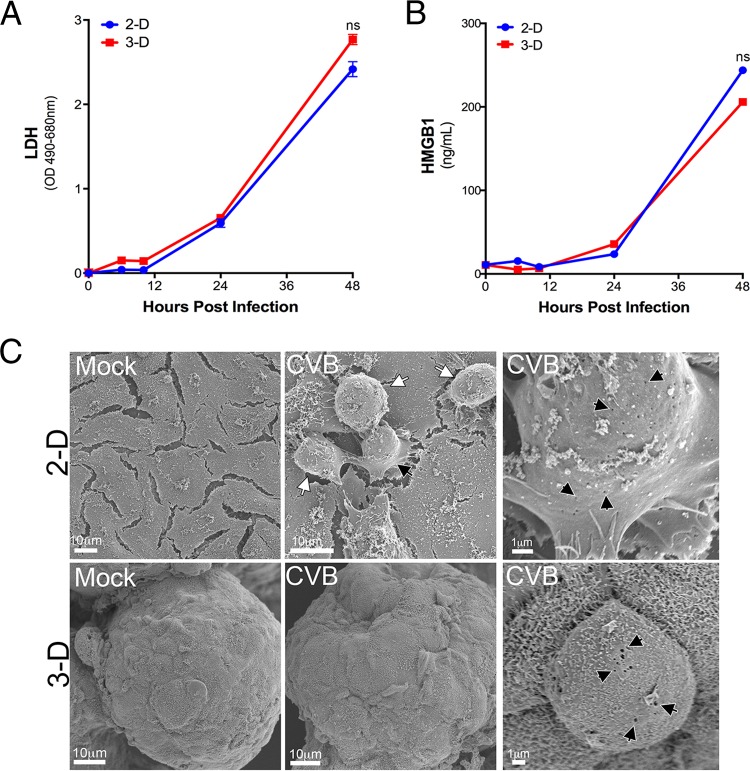
(A) Levels of released LDH in 2-D or 3-D cultures of Caco-2 cells infected with CVB (1 PFU/cell) for the indicated times postinfection. OD 490-680 nm, optical density at 490 to 690 nm. (B) Levels of HMGB1 (in nanograms per milliliter) in the supernatants of 2-D or 3-D cultures of Caco-2 cells infected with CVB (1 PFU/cell) for the indicated times. Values that are not significantly different (ns) as determined by a Student’s *t* test are indicated. (C) Scanning electron micrographs of 2-D or 3-D cultures of Caco-2 cells. Mock-infected controls and cultures infected with CVB for 24 h are shown. (Right) Zoomed-in images of single infected cells with black arrowheads denoting membrane lesions.

Enteroviruses primarily egress by direct cell death-mediated lysis of the host cell membrane. Because the release of CVB from polarized IECs is dependent on CVB-induced necrotic cell death (17), we analyzed cell cytotoxicity to determine whether the difference in CVB release between 2-D and 3-D cultures resulted from differences in cell death. To do this, we first measured the levels of released lactate dehydrogenase (LDH) in the supernatants of 2-D and 3-D cultures of CVB-infected Caco-2 cells and found that the levels were comparable between cell culture conditions (Fig. 6A). In addition, we measured the levels of high-mobility-group box 1 (HMGB1), which is released into the cell culture supernatants of cells undergoing necrosis (45), in the supernatants of infected cultures given that Caco-2 cells primarily undergo necrosis in response to CVB infection (17) and found near-equivalent levels of HMGB1 released from both 2-D and 3-D culture conditions (Fig. 6C). Finally, we found that both 2-D and 3-D cultures of CVB-infected cells exhibited significant morphological changes as assessed by SEM (Fig. 6C, right panels), such as cell rounding and the appearance of membrane lesions characteristic of necrosis (Fig. 6C). Collectively, these data suggest that the increased extracellular CVB titers in 3-D Caco-2 cultures did not result from any differences in cell death or cytotoxicity.

### Transcriptional profiling between 2-D and 3-D cultures of CVB-infected Caco-2 cells by RNA-Seq.

**FIG 7:**
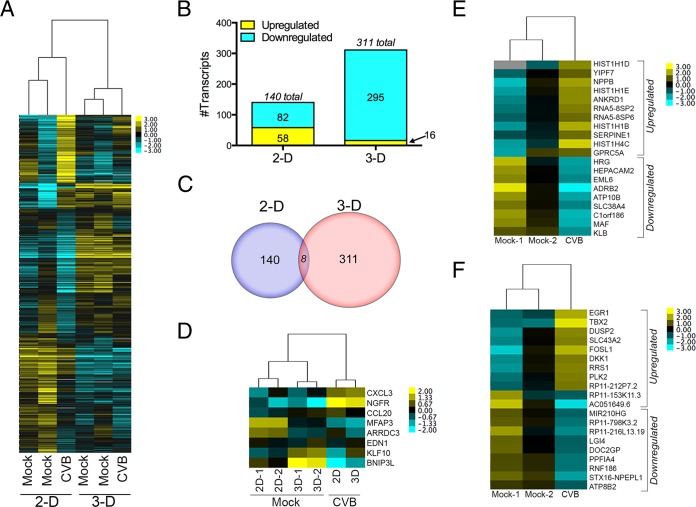
(A) Hierarchical clustering heat map of genes expressed in mock- or CVB-infected 2-D or 3-D cultures of Caco-2 cells as determined by RNA-Seq. The heat map shows the data from two independent mock-infected 2-D or 3-D cultures. (B) Number of differentially expressed genes from a single CVB-infected culture. The number of differentially expressed genes was determined by DESeq2 (*P* < 0.01) in 2-D or 3-D cultures of mock- versus CVB-infected cells. The number of upregulated transcripts is shown in yellow, and the number of downregulated transcripts is shown in blue. (C) Venn diagram of number of differentially expressed transcripts (as determined by DESeq2; *P* < 0.01) between 2-D and 3-D cultured Caco-2 cells. Only eight transcripts were shared between the two culture conditions. (D) Hierarchical clustering heat map of genes differentially expressed (*P* < 0.01) and shared between 2-D and 3-D cultures of CVB-infected Caco-2 cells as determined by RNA-Seq, followed by DeSeq2 analysis. (E) Hierarchical clustering heat map of genes differentially expressed (*P* < 0.01) specifically in CVB-infected 2-D cultures as determined by RNA-Seq, followed by DeSeq2 analysis. (F) Hierarchical clustering heat map of genes differentially expressed (*P* < 0.01) specifically in CVB-infected 3-D cultures as determined by RNA-Seq, followed by DeSeq2 analysis. In panel A and panels D to F, the intensity of the color indicates the level of gene expression (yellow for upregulation and blue for downregulation), and gray indicates that no RNA-Seq reads were detected for that transcript in that sample

We next profiled transcriptional changes between 2-D and 3-D cultures of Caco-2 cells infected with CVB to determine whether alterations in gene expression could account for the differences in CVB release between the culture conditions. To do this, we utilized RNA-Seq followed by DESeq2 analysis to identify genes differentially expressed between mock- and CVB-infected cultures. We observed significant changes in gene expression upon CVB infection of either 2-D or 3-D Caco-2 cell cultures (Fig. 7A; see Data Sets S2 and S3 in the supplemental material). CVB infection induced significant (*P* < 0.01) changes in the expression of 140 transcripts in 2-D cultures and 311 transcripts in 3-D cultures (Fig. 7B). In 2-D cultures, there were 58 genes upregulated in response to CVB infection and 82 genes downregulated (Fig. 7B). In contrast, the vast number of genes differentially expressed in CVB-infected 3-D cultures were downregulated (295 of 311 total genes) (Fig. 7B). Interestingly, of the transcripts differentially expressed in 2-D and 3-D cultures of CVB-infected cells, only 8 were common to both cell culture conditions (Fig. 7C). These included induced genes such as the chemokines CCL20 (CC chemokine ligand 20) and CXCL3 (CXC chemokine ligand 3), the arrestin family member arrestin domain-containing 3 (ARRDC3), nerve growth factor receptor (NGFR), and endothelin 1 (EDN1) (Fig. 7C). Only a single gene, BCL2/adenovirus E1B 19-kDa-interacting protein 3-like (BNIP3L)/NIX, which is a proapoptotic mitochondrion-localized homolog of NIP3 (46), was downregulated in both 2-D and 3-D cultures of CVB-infected cells (Fig. 7D). In addition, a single gene, which mapped to a long noncoding RNA (lncRNA) (RP11-563J2.2) was differentially modulated between 2-D and 3-D CVB-infected cell cultures, with an upregulation in 2-D infected cell cultures and a corresponding downregulation in 3-D infected cell cultures (Data Sets S2 and S3).

10.1128/mSphere.00030-15.3 Data Set S2  Spreadsheet of DeSeq2 analysis of RNA-Seq studies of 2-D cultures of mock- or CVB-infected Caco-2 cells. The gene symbols, log_2_ fold changes (green), and *P* values (red) are shown. Download Data Set S2, XLSX file, 0.1 MB. Copyright © 2015 Drummond et al. 2015 Drummond et al. This content is distributed under the terms of the Creative Commons Attribution 4.0 International license.

10.1128/mSphere.00030-15.4 Data Set S3  Spreadsheet of DeSeq2 analysis of RNA-Seq studies of 3-D cultures of mock- or CVB-infected Caco-2 cells. The gene symbols, log_2_ fold changes (green), and *P* values (red) are shown. Download Data Set S3, XLSX file, 0.1 MB. Copyright © 2015 Drummond et al. 2015 Drummond et al. This content is distributed under the terms of the Creative Commons Attribution 4.0 International license.

The majority of genes differentially induced/suppressed in response to CVB infection were unique to 2-D or 3-D cultures. In 2-D cultures of infected cells, this included the induction of specific genes such as YIP1 family member 7 (YIPF7), a member of the YIP family of Golgi complex-localized components (47) that have been associated with intestinal inflammation (48) and the tumor necrosis factor alpha (TNF-α)-inducible ankyrin repeat domain 1 (ANKRD1) among others (Fig. 7E; see Data Set S2 in the supplemental material). Pathway analysis of genes differentially expressed in 2-D cultures of CVB-infected cells revealed an enrichment in NF-κB activation pathways (*P* = 4.02e^−8^), immune response to tumor necrosis factor receptor 2 (TNF-R2) signaling pathways (*P* = 1.62e^−8^), and antiapoptosis and survival signaling (*P* = 3.13e^−4^) (Data Set S4). In 3-D cultures of CVB-infected cells, unique differentially induced genes included the secreted Wnt antagonist Dickkopf-1 (DKK1), which positively regulates proliferation of the intestinal epithelium whose expression thus correlates with decreased cell proliferation and differentiation (49) and the transcriptional regulator’s early growth response 1 (EGR1) and T-box protein 2 (TBX2) (Fig. 7F and Data Set S3) among others. Pathway analysis of genes differentially expressed in CVB-infected 3-D cultures revealed an enrichment in the Wnt signaling pathway (*P* = 6.09e^−3^), differentiation of gastric mucosa (*P* = 2.36e^−3^), and immune response C3a signaling (*P* = 4.61e^−3^) (Data Set S5).

10.1128/mSphere.00030-15.5 Data Set S4  Spreadsheet of pathway analysis (conducted in MetaCore from GeneGo) of pathways enriched in 2-D cultures of CVB-infected cells. The pathways significantly (*P* < 0.001) enriched and the numbers of transcripts in the data set enriched in a given pathway are shown. Download Data Set S4, XLS file, 0.05 MB. Copyright © 2015 Drummond et al. 2015 Drummond et al. This content is distributed under the terms of the Creative Commons Attribution 4.0 International license.

10.1128/mSphere.00030-15.6 Data Set S5  Spreadsheet of pathway analysis (conducted in MetaCore from GeneGo) of pathways enriched in 3-D cultures of CVB-infected cells. The pathways significantly (*P* < 0.001) enriched and the numbers of transcripts in the data set enriched in a given pathway are shown. Download Data Set S5, XLS file, 0.05 MB. Copyright © 2015 Drummond et al. 2015 Drummond et al. This content is distributed under the terms of the Creative Commons Attribution 4.0 International license.

### CVB infection in 3-D cultures of HeLa cells and PV infection in 3-D cultures of Caco-2 cells.

**FIG 8:**
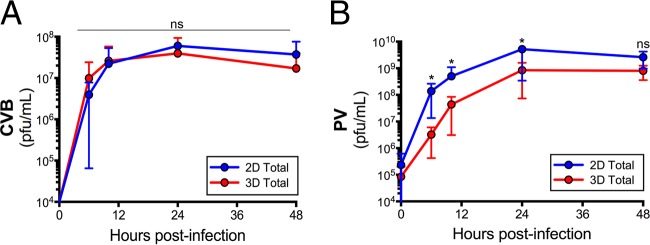
(A) CVB titers (in PFU/milliliter) of virus collected from the medium of 2-D or 3-D cultures of HeLa cells infected with CVB (1 PFU/cell) for the indicated hours postinfection. (B) PV titers (in PFU/milliliter) of virus collected from the medium of 2-D or 3-D cultures of Caco-2 cells infected with PV (3 PFU/cell) for the indicated hours postinfection. Data are shown as means ± standard deviations and are averaged from three independent STLVs. Values that are significantly different (*P* < 0.05) by a Student’s *t* test are indicated by an asterisk. Values that are not significantly different (ns) by a Student’s *t* test are also indicated.

Because we observed differences in CVB infection between 2-D and 3-D cultures of Caco-2 cells, we next assessed whether these differences would occur in 3-D cultures of other cell types, such as HeLa cells. Whereas our results in 3-D Caco-2 cultures pointed to an enhanced release of CVB from these cultures, we found that CVB infection, as assessed by intracellular and extracellular titers in 2-D and 3-D cultures of HeLa cells, were near equivalent between both culture conditions (Fig. 8A). In addition, we found that, whereas 3-D cultures of Caco-2 cells released more infectious virus than 2-D cultures did, cells cultured in 3-D became more resistant to infection by PV. Importantly, this was not the result of alterations in the expression of PVR, which were not significantly different between 2-D and 3-D cultures (see Fig. S1 in the supplemental material). Taken together, these data point to the cell type- and virus type-specific nature of the release of more infectious virus from 3-D Caco-2 cell cultures.

## DISCUSSION

Here, we describe the development of a 3-D-based culture system using Caco-2 cells that can be applied to the study of enterovirus infection of human IECs. We show that Caco-2 cells cultured in the RWV bioreactor develop morphological and transcriptional phenotypes more similar to the GI epithelium *in vivo*. In addition, we show that these cells can be infected by CVB and release more infectious virus than 2-D cells at early stages of the viral life cycle.

Much of what we know regarding the interactions between CVB and polarized IECs has been generated using cell lines, such as Caco-2 cells, under standard 2-D culture conditions. While these studies have provided important insights into aspects of CVB infection of polarized IECs, they are inherently limited by the significant differences that exist between cell culture and *in vivo* systems. Although the 3-D system we describe here develops phenotypes resembling the GI epithelium *in vivo*, it is not an absolute model of the GI tract *in vivo*, which has the added complexity of other cell types, including immune components as well as a bacterial microbiome that undoubtedly influences a variety of aspects of viral pathogenesis. Indeed, previous studies of oral PV infections in human PVR transgenic mice lacking expression of the IFN-α/β receptor suggest that the microbiome facilitates PV infection of the GI epithelium (50, 51). However, given the need to ablate the type I IFN system to allow for oral infection in mice and the fact that humans are the primary hosts for enteroviruses, the development of human-based systems to better model enterovirus-IEC interactions is critical. Thus, the system we describe here provides a platform by which to study CVB, and other enterovirus, infection of the GI epithelium and is likely to provide insights into the dialogue that exists between the virus and IECs. Because this system is based on a cell line, it also has the advantage of being more easily manipulated genetically than other models (such as small animals) and can thus be utilized for gene depletion and/or knockdown studies by techniques such as approaches based on RNA interference (RNAi) or the clustered regularly interspaced short palindromic repeat (CRISPR) Cas9.

A central question that has remained unanswered in the field of CVB-polarized IEC interactions is the mechanism by which the virus attaches to DAF on the complex differentiated apical surfaces of IECs and circumnavigates this barrier to reach CAR in the TJ. Given that studies of CVB entry into polarized IECs have been restricted to 2-D culture conditions (13, 14), which exhibit a less complex apical surface than their 3-D cell counterparts, these questions are inherently more difficult to fully address. Our work presented here suggests that CVB is adept at entering the GI epithelium rapidly, as we found that entry occurred with similar kinetics between 2-D and 3-D cultured cells, despite the complex nature of the apical surface of Caco-2 cells cultured in 3-D. In contrast, our studies suggest that PV is either less efficient at entering IECs or that viral replication is less efficient in 3-D, given that we observed a significant reduction in PV titers in Caco-2 cells cultured in 3-D. In the case of both CVB and PV, receptor expression in 2-D and 3-D cultures are near equivalent (see Fig. S1 in the supplemental material), thus the differences in viral infection between 2-D and 3-D cells cannot be due to receptor expression alone, although receptor localization may certainly play a role.

Despite producing lower levels of vRNA and newly synthesized viral proteins and generating near-equivalent intracellular CVB titers, we found that 3-D cultures of Caco-2 cells released more infectious virus than cells cultured in 2-D at early stages of the viral life cycle did. As we did not detect any differences in CVB-induced cell death or membrane destruction between 2-D and 3-D cultures, it is difficult to reconcile how 3-D cultures are more efficient at viral release. Although cell death and enhanced membrane leakage are likely to be the primary mechanisms of enteroviral egress, two additional mechanisms recently proposed suggest that enteroviruses can also be released in cell-derived microvesicles (52) and/or by a nonlytic release mechanism (53). While we cannot exclude the possibility that some amount of released CVB in 3-D Caco-2 cell cultures resides in microvesicles, we found that >99% of the viral activity of CVB-infected supernatants of 2-D and 3-D cultures could be inhibited by an anti-CVB neutralizing antibody (see Fig. S2 in the supplemental material). However, previous work on vesicle-associated hepatitis A virus (HAV) showed that some antibodies neutralize this form of the virus postentry (54), although the mechanism by which this occurs remains unclear.

10.1128/mSphere.00030-15.7 Figure S2  Supernatants of 2-D or 3-D cultures of Caco-2 cells infected with CVB, or CVB virus stock, were incubated with a control antibody or anti-CVB neutralizing antibody (clone 280-5F-4E-5E; Millipore) at a 1:600 dilution for 1 h and then added to HeLa cells for 6 h. Infection was quantified by RT-qPCR and is shown as a percentage of the level for the 2-D supernatant with control antibody controls. Download Figure S2, TIF file, 0.1 MB. Copyright © 2015 Drummond et al. 2015 Drummond et al. This content is distributed under the terms of the Creative Commons Attribution 4.0 International license.

The nonlytic release of PV has been proposed to occur via a process facilitated by autophagy (53), which is also associated with the formation of enterovirus-induced replication organelles (55). Similar to other GI-derived cancer cell lines, Caco-2 cells exhibit high rates of resting autophagy (56, 57), which are reduced upon differentiation (58). In the normal GI epithelium *in vivo*, autophagy is also active and is upregulated in proliferating and progenitor cells (58). Given the high degree of association between autophagy and the GI epithelium, it is possible that the enhanced release of CVB from 3-D cultures of infected Caco-2 cells is facilitated by alterations in the rate of autophagy in select subpopulations of cells, and thus the enhanced release of viral particles by a nonlytic mechanism. Thus, it is possible that the enhanced titers of released CVB early in infection in 3-D cultures may be the result of several parallel pathways, which might include nonlytic release in either microvesicles or by an autophagy-mediated pathway.

Collectively, our studies show that Caco-2 cells grown in the RWV bioreactor may provide a cell culture model that structurally and transcriptionally represents tissue of the human GI tract and provides a tool to improve our understanding of enterovirus-host interactions in polarized IECs.

## MATERIALS AND METHODS

### Cell culture.

Caco-2 cells (ATCC clone HTB-37) were cultured in modified Eagle’s medium with 10% fetal bovine serum, nonessential amino acids, penicillin-streptomycin, and sodium pyruvate. HeLa cells (CCL-2) were grown in modified Eagle’s medium with 5% fetal bovine serum, nonessential amino acids, penicillin-streptomycin, and sodium pyruvate.

### Rotating wall vessel bioreactor cultures.

For 3-D culturing, Caco-2 or HeLa cells were grown in the slow-turning lateral vessel (STLV) (Synthecon Inc.) bioreactor system, based on previously established protocols (26, 31, 32). Cells were grown to confluence in standard 2-D flasks and removed with 0.05% trypsin-EDTA, enumerated using a TC20 automated cell counter (Bio-Rad), and combined with 250 mg Cytodex-3 beads (Sigma Aldrich) in 55 ml of complete medium. The bead/cell mixture was then added to a sterile STLV and incubated at 37°C under static conditions for 1 h before attachment to the 4H rotary cell culture system (Synthecon Inc.). The reactor was rotated at a speed of 20 rpm within a humidified incubator at 37°C and 5% CO_2_ for the duration of the culture period. The culture medium was replaced 5 days after the initial STLV seeding and every 2 days thereafter. Cell-covered beads were removed for analysis or infection on day 21, unless otherwise stated and transferred to 24-well tissue culture plates for infection and subsequent experiments. To calculate cell number per volume of beads, cells were removed with 0.05% trypsin-EDTA at 37°C and enumerated as described above. In parallel, control 2-D cells from monolayers were also seeded into 24-well plates. In both cases, 4 × 10^5^ cells were seeded per well.

### Viruses and plaque assays.

Experiments were performed with CVB3-RD or PV, expanded as described previously (59, 60) with 1 to 3 PFU/cell. For all infections, virus was adsorbed to cells for 1 h at 16°C, followed by removal of unbound virus by washing with phosphate-buffered saline (PBS). Complete cell medium was then added, and cells were incubated at 37°C throughout the period of infection. Samples were collected at the indicated times. For plaque assays, CVB-infected Caco-2 or HeLa cells were harvested at the indicated times by cell scraping. In parallel, supernatants were collected to quantify extracellular CVB titers. Samples were freeze-thawed three times, and viral titers were determined by plaque assays as described previously (17).

### Immunofluorescence microscopy.

Cells were washed with PBS and fixed with 4% paraformaldehyde or with ice-cold 100% methanol followed by permeabilization with 0.25% Triton X-100 in PBS and incubation with the indicated primary antibodies for 1 to 2 h at room temperature. After the cells were washed, they were incubated with secondary antibodies for 30 min at room temperature, washed, and mounted with Vectashield (Vector Laboratories) containing 4′,6-diamidino-2-phenylindole (DAPI). Images were captured using an FV1000 confocal laser scanning microscope (Olympus) and contrasted and merged using Adobe Photoshop. Antibodies or other reagents for fluorescence microscopy were as follows: mouse anti-enterovirus VP1 (NC-ENTERO; Leica), mouse anti-ZO-1 (mid region; Invitrogen), mouse antiezrin (Millipore), rabbit antioccludin (N-terminal region; Invitrogen), mouse anti-β-catenin (Invitrogen), mouse anti-GLUT5 (Sigma), rabbit anti-E-cadherin (Invitrogen), rabbit anti-glyceraldehyde-3-phosphate dehydrogenase (anti-GAPDH) (Santa Cruz Biotechnology), and FITC-conjugated *Ulex europaeus* agglutinin I (UEA1; Sigma). Rabbit anti-CAR (45) and mouse anti-DAF IF7 were kindly provided by Jeffrey Bergelson, Children’s Hospital of Philadelphia. Alexa Fluor-conjugated secondary antibodies were purchased from Invitrogen.

### Electron microscopy.

Cells were fixed in 2.5% glutaraldehyde, washed with PBS, and postfixed in aqueous 1% OsO4. After the fixed cells were washed with PBS, samples were dehydrated through a graded ethanol series (30% to 100%) and washed with absolute ethanol before drying in hexamethyldisilizane solution, followed by air drying. For 3-D cultures, beads were picked up with double-sided copper tape. The cells were subsequently embedded in Epon resin and thin sectioned for imaging utilizing a JEOL 1011 transmission electron microscope or subjected to critical point drying and mounted on aluminum stubs for imaging with a scanning electron microscope (JSM 6330F).

### Immunoblotting.

Protein lysates were collected in radioimmunoprecipitation assay (RIPA) buffer (50 mM Tris-HCl, 1% NP-40, 0.25% sodium deoxycholate, 150 mM NaCl, 1 mM EDTA) containing a protease inhibitor cocktail (Promega). Lysates were separated on 4 to 20% gradient Tris-HCl SDS-polyacrylamide gels, transferred to nitrocellulose membranes, and blocked for 1 h in PBS containing (0.5%) Tween 20 (PBST) and 5% milk. After the membranes were washed, they were incubated with anti-rabbit or anti-mouse antibodies conjugated to IRDye 680LT or 800CW and visualized with the Odyssey infrared imaging system.

### RNA-Seq.

Total RNA was extracted using GenElute mammalian total RNA miniprep kit (Sigma) according to the manufacturer’s protocol. RNA samples were treated with RNase-free DNase (Sigma). RNA integrity was assessed by NanoDrop, Qubit assay, and/or using an Agilent 2100 bioanalyzer per each manufacturer’s specifications. Sample amounts were normalized, and 1,000 ng was used for library preparation using the NEB Ultra RNA Library Preparation kit per the manufacturer’s instructions. Library quality control (QC) and quantitation were performed on all individual libraries by the Qubit assay and by using the Agilent 2100 bioanalyzer. Libraries were normalized and pooled via Qubit measurement. The final pool was quantitated via quantitative PCR (qPCR). Sequencing was performed on the Illumina HiSeq2500 rapid-run mode on one flow cell (two lanes) per the system manufacturer. Raw RNA-seq data were processed, normalized, and mapped to the human reference genome (hg19) using CLC Genomics Workbench 8 (Qiagen). Differentially expressed genes were identified using DESeq2 (38) with the indicated significance cutoffs. Hierarchical clustering was performed using Cluster 3.0/Java Treeview and heat maps were generated using MeViewer software (17).

Pathway analysis was performed using MetaCore by GeneGo, with a statistical cutoff of *P* < 0.001 applied for pathway enrichment.

### Quantitative PCR.

Total RNA was extracted using GenElute mammalian total RNA miniprep kit (Sigma) according to the manufacturer’s protocol, treated with RNase-free DNase (Sigma), and reverse transcribed using iScript cDNA synthesis kit (Bio-Rad). For each sample, 1 µg total RNA was used for cDNA synthesis. RT-qPCR was performed using iQ SYBR green supermix (Bio-Rad) in an Applied Biosystems StepOne real-time PCR machine. Gene expression was calculated using a modified Δ*C_T_* method (*C_T_* stands for threshold cycle) based upon normalization to human actin. Primer sequences can be found in Table S1 in the supplemental material.

10.1128/mSphere.00030-15.8 Table S1  Primer sequences used for RT-qPCR studies. Download Table S1, TIF file, 0.2 MB. Copyright © 2015 Drummond et al. 2015 Drummond et al. This content is distributed under the terms of the Creative Commons Attribution 4.0 International license.

### Neutral red assay.

Neutral red containing CVB particles (NR-CVB) were prepared as described previously (16, 61). To synchronize infections, NR-CVB (10 PFU/cell) were adsorbed to cells at 16°C for 1 h prior to incubation in dark conditions at 37°C in a humidified incubator. Following infection for 0 to 3 h, cells were illuminated for 20 min with a light box. The cells were exposed to light for 20 min or kept in semidark conditions for the duration of the infection (~18 h). Infection was quantified by RT-qPCR as described above.

### DAF immunoblocking assay.

Cells were preincubated with anti-DAF IF7 at a dilution of 1:50 or with an isotype control antibody for 1 h prior to CVB infection, as described previously (6). Cells were then infected with CVB (1 PFU/cell) for ~5 h, and infection was quantified by RT-qPCR as described above.

### HMGB1 enzyme-linked immunosorbent and lactose dehydrogenase release assays.

Cellular supernatants were collected from 2-D and 3-D cultures of Caco-2 cells at 0, 6, 10, 24, and 48 h postinfection. The levels of released HMGB1 were measured utilizing an HMGB1 enzyme-linked immunosorbent assay (ELISA) kit (IBL-International) per the manufacturer’s instructions. The levels of released LDH in cellular supernatants were measured using the LDH cytotoxicity assay kit (Pierce) per the manufacturer’s protocol.

### Statistics.

All statistical analysis was performed using GraphPad Prism, unless otherwise noted. Student’s *t* tests were performed unless otherwise noted in the figure legends.
