# Supplementary figures and images for: A Three-Dimensional Cell Culture Model To Study Enterovirus Infection of Polarized Intestinal Epithelial Cells
Source: mSphere. 2015 Nov 18;1(1):e00030-15. doi: 10.1128/mSphere.00030-15 (PMC4863623; doi:10.1128/mSphere.00030-15)

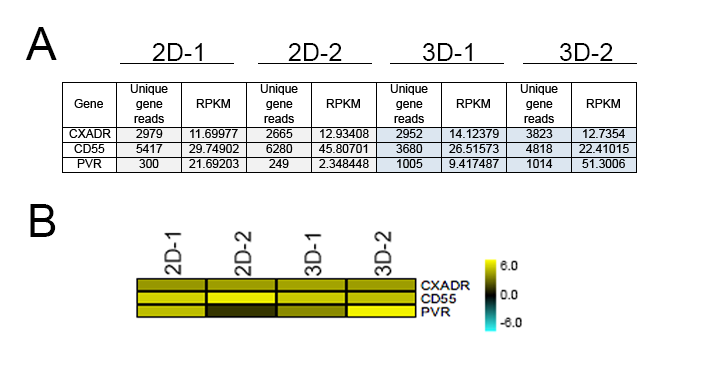

Supplement: Figure S1 [file sph001160030sf2.tif]

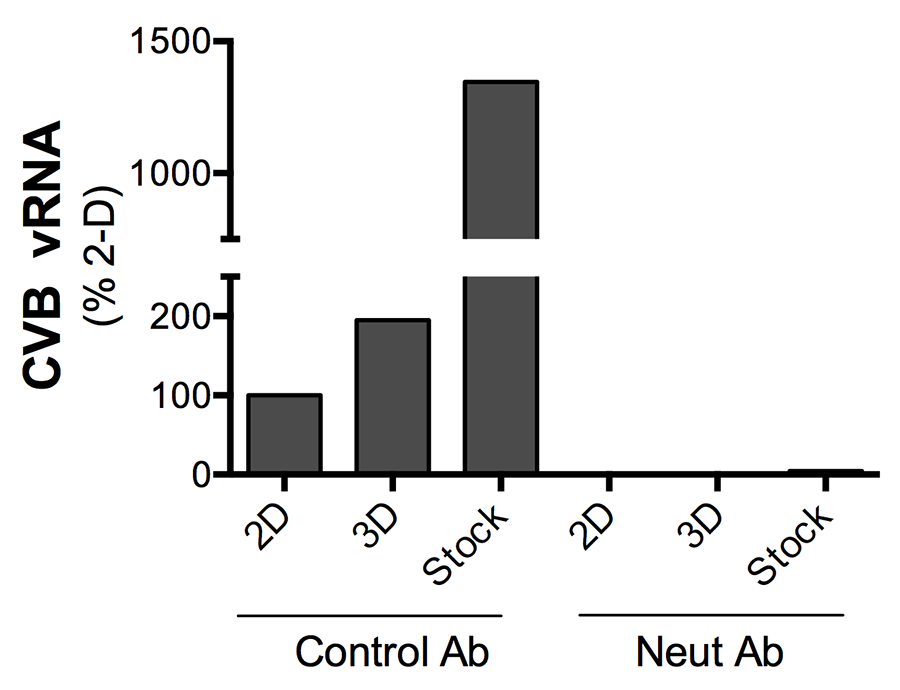

Supplement: Figure S2 [file sph001160030sf3.tif]

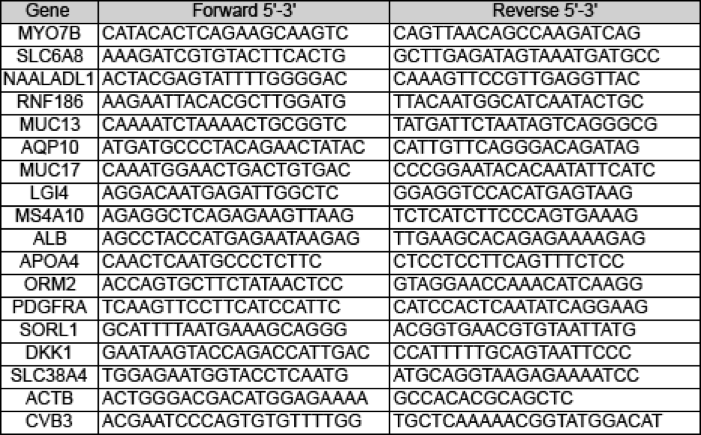

Supplement: Table S1 [file sph001160030st1.tif]
